# Burden of refractory and unexplained chronic cough on patients’ lives: a cohort study

**DOI:** 10.1183/23120541.00425-2023

**Published:** 2023-09-25

**Authors:** Luis Puente-Maestu, Ignacio Dávila, Santiago Quirce, Astrid Crespo-Lessmann, Eva Martínez-Moragón, Javier Sola, María Luisa Nieto, Francisco Javier González-Barcala, Luis Cea-Calvo, Marta Sánchez-Jareño, Cristina Rivas-Pardinas, Christian Domingo

**Affiliations:** 1 Servicio de Neumología, Hospital Universitario Gregorio Marañón, UCM, Madrid, Spain; 2 Servicio de Alergia, Hospital Universitario de Salamanca, Departamento de Ciencias Biomédicas y del Diagnóstico, Facultad de Medicina, Universidad de Salamanca, Salamanca, Spain; 3 Servicio de Alergia, Hospital Universitario La Paz, Madrid, Spain; 4 Servicio de Neumología y Alergia, Hospital de la Santa Creu i Sant Pau, Barcelona, Spain; 5 Servicio de Neumología, Hospital Universitario Doctor Peset, Valencia, Spain; 6 Servicio de Alergia, Hospital Universitario Ramón y Cajal, Madrid, Spain; 7 Servicio de Neumología, Hospital Arnau de Vilanova, Valencia, Spain; 8 Grupo de Investigación Traslacional en Enfermedades de las Vías Aéreas (TRIAD); Instituto de Investigación Sanitaria de Santiago de Compostela (IDIS); Departamento de Medicina, Universidad de Santiago de Compostela; Departamento de Medicina Respiratoria, Hospital Universitario de Santiago de Compostela, Santiago de Compostela, Spain; 9 Medical Affairs, MSD, Madrid, Spain; 10 Servicio de Neumología, Corporació Parc Taulí, Universitat Autònoma de Barcelona (UAB), Sabadell, Spain

## Abstract

**Background:**

Chronic cough (cough lasting for ≥8 weeks) can lead to significant impairment in quality of life (QoL). Using patient-reported outcomes, this cohort study assessed the perceived impact of chronic cough on QoL and everyday life in patients from outpatient hospital clinics with refractory chronic cough (RCC) or unexplained chronic cough (UCC).

**Methods:**

This was a multicentre, non-interventional survey study. Cough severity was assessed on a 0−100 mm Visual Analogue Scale (VAS). Frequency, intensity and disruptiveness of cough were assessed using an adaptation of the Cough Severity Diary. The impact of cough on QoL was assessed using the Leicester Cough Questionnaire (LCQ). The physical impact of cough and associated impact on everyday life activities were explored using purpose-designed questions.

**Results:**

191 patients responded to the survey; 121 (63.4%) had RCC and 149 were women (78.0%). Mean score on the cough severity VAS was 62.9 mm. Mean LCQ total score of 11.9 indicated reduced QoL. Cough impaired patients’ everyday life, including the inability to speak fluently (58.0% of patients) and feeling tired/drained (46.6%). Women perceived poorer chronic cough-related QoL than men, as reflected by lower LCQ scores, and greater impairment of physical health, including cough-related stress urinary incontinence, and psychological health.

**Conclusions:**

Patients with RCC/UCC experience a significant burden in their everyday life, including impaired QoL, and perceive a negative impact on physical and psychological health and everyday activities, affecting work, relationships and leisure activities. The impact appears to be greater in women than men for several of the aspects studied.

## Tweetable abstract

**Refractory and unexplained chronic cough significantly impair patients’ quality of life, impacting their physical and psychological health, and everyday life. The impact appears to be stronger in women than men.** https://bit.ly/3Yu8Rgg

## Introduction

In adults, chronic cough is defined as daily cough lasting for ≥8 weeks [1] and is a common medical condition. Recent web-based National Health and Wellness Surveys estimate the prevalence of chronic cough in the previous 12 months at ≈5% in Japan [2], Germany [3], the United States [4] and Spain [5]. The estimated lifetime prevalence of chronic cough in Spain is 8.2%, affecting around 3.3 million adults [5].

Chronic cough can be caused by diseases such as gastro-oesophageal reflux disease (GORD), asthma, eosinophilic bronchitis, postnasal drip syndrome or rhinosinusitis, COPD, pulmonary fibrosis and bronchiectasis, and by environmental or behavioural factors, such as exposure to cigarette smoke or environmental pollutants, especially particulates [6]. However, because not all individuals with these conditions or environmental trigger exposure develop chronic cough, it is considered that other factors are at play. Persistent chronic cough can be categorised as refractory chronic cough (RCC) or unexplained chronic cough (UCC) [7]. RCC is defined as a cough that persists despite adherence to optimal guideline-based treatment for the underlying condition. UCC is a diagnosis of exclusion that refers to circumstances where no cause for cough can be determined [8]. However, the 2020 European Respiratory Society guidelines on diagnosis and treatment of chronic cough introduced the term chronic refractory cough to cover situations, particularly among adults, where cough persists despite thorough investigation and treatment of cough-associated conditions according to clinical practice guidelines [9]. This terminology emphasises the concept of chronic cough as an entity itself and the treatment of patients according to different phenotypes or treatable traits. From this point of view, RCC and UCC are regarded as the same entity, with hypersensitivity to the cough reflex as the common feature [1, 9]. Hypersensitivity to the cough reflex is characterised by dry coughing in response to low level exposure to thermal, mechanical or chemical stimuli [10]. The distinct pathophysiological process is thought to be due to increased activation or excitability of airway sensory neuron receptors to stimuli and/or by an increased response of the central nervous system cough network to the central transmission of inputs [7, 11–13].

Multiple studies have indicated that chronic cough impacts on physical, psychological and social measures with consequent impairment of patients’ daily life activities and quality of life (QoL) [14–24]. In a large cross-sectional multinational European internet survey, 96% of 1120 respondents with chronic cough considered that cough negatively affected their QoL and 81% indicated that it limited their ability to undertake activities [19].

In Spain, data are lacking about the impact of cough specifically in patients with RCC or UCC. To study these groups, an observational study was undertaken of RCC/UCC patients who attended outpatient pulmonology and allergy clinics in Spanish hospitals. Recently, we reported high health resource utilisation in Spanish hospitals due to RCC/UCC (burden to the healthcare system) [25]. Herein, as a companion article, we report the impact of chronic cough on patients’ QoL and everyday lives (burden to patients).

## Methods

This multicentre, non-interventional study of a cohort of patients with RCC/UCC was conducted with the participation of allergy and pulmonology outpatient hospital clinics from the Spanish National Healthcare System. The focus of the study was on disease characteristics, diagnostic procedures, cough management and the impact of cough on patients’ everyday life. Information on disease history and cough management, as well as comorbidities and cough-related diseases (*i.e.* GORD, asthma, eosinophilic bronchitis, postnasal drip syndrome or upper airway cough syndrome), was collected retrospectively from patients’ existing clinical charts [25], all diagnoses having been made based on clinical guideline recommendations and practice. Information about the impact of chronic cough on QoL and patients’ everyday life was collected through a parallel cross-sectional survey completed by patients.

### Patient population

Consecutive patients with RCC or UCC who were seen at clinics between November 2020 and June 2022 were invited by their pulmonologist or allergist to participate in the study. Inclusion criteria were adults (>18 years old) with a diagnosis of RCC or UCC reflected in clinical chart review, or according to the treating physician's judgement after reviewing the clinical history, diagnostic tests and therapies used to treat chronic cough in the past. Patients had to have chronic cough of >1 year's duration (*i.e.* had been attending the outpatient clinic for chronic cough at least 1 year before the enrolment date) and to have cough on the day of study enrolment. Exclusion criteria included smoking or having stopped smoking in the previous 12 months; current treatment with angiotensin-converting enzyme (ACE) inhibitors; chronic cough related to COPD, cancer, active infection, bronchiectasis, interstitial lung disease, cystic fibrosis or Gilles de la Tourette syndrome; current participation in interventional studies; or suffering conditions that, in the judgement of the treating physician, advised against participation (*e.g.* cognitive impairment, major depression, end-stage disease).

### Procedures

Patients completed a printed survey which consisted of validated questionnaires and *ad hoc* questions about the impact of cough on QoL and daily life, including everyday activities, as well as professional, relationship and leisure activities. Patients completed the survey without the intervention of attending physicians and/or investigator participation or overview. Clinical teams were allowed to clarify any items patients did not understand but were instructed not to guide or check patients’ responses. Completed surveys were placed in an envelope and sealed.

Cough severity, as perceived by patients, was measured using a Visual Analogue Scale (VAS) validated for use in patients with RCC and UCC [26]. On a horizontal line 100 mm in length, patients indicated the severity of cough experienced during the previous day with scores ranging from 0 (no cough) to 100 (worst cough, unbearable symptoms).

Patients’ perceptions of the frequency, intensity and disruptiveness of cough were assessed using an adaptation of the 7-item Cough Severity Diary (CSD) validated in the Spanish language, where each item is rated on an 11-point Likert scale, with higher scores indicating greater severity [27, 28]. The CSD is designed to be completed at the end of the day; however, because most patients would complete it during a morning clinic appointment, questions were adapted to refer to the previous day. For this reason, CSD scores were not calculated, and only patient responses are described.

The impact of cough on QoL was assessed using the Leicester Cough Questionnaire (LCQ) [29], which has been validated for use in patients with RCC or UCC [30]. A validated version in the Spanish language was used in this study. This 19-item cough-specific health-related QoL questionnaire comprises three domains (physical, psychological and social). Patients assess their cough symptoms and the impact of cough on QoL over the previous 2 weeks, using a 7-point Likert-type scale for each item. Mean scores of each domain are calculated as the average of their items, yielding values from 1 to 7, and the LCQ total score is the sum of the three individual mean scores, ranging from 3 to 21. Higher LCQ scores indicate better QoL [29].

The physical impact of cough and its impact on everyday life activities were explored using questions developed based on previous publications [15] or *ad hoc* for this study with the participation of experts in chronic cough. Patients were asked to respond to six questions about the physical impact of cough using a 5-point Likert scale with categories ranging from 1 (never/hardly ever) to 5 (mostly/always), and to respond to 15 questions about the impact of chronic cough on aspects of everyday life using a 7-point Likert scale with categories ranging from 1 (not at all) to 7 (extremely high). For these items, patients were prompted to provide an assessment not limited to a specific time period but, rather, considering how cough had been impacting on their lives in general.

### Statistical analysis

This non-interventional study had primarily exploratory objectives and no prespecified hypothesis. A sample size of 196 patients was calculated based on a conservative approach with 95% confidence and 7% precision for an expected prevalence of 50% of any categorical variable. No stratification was required between RCC and UCC groups or between allergy and pulmonology clinics.

VAS item and total scores and LCQ domain and total scores are presented as mean±sd. Proportions of patients who assigned the highest scores to individual items (greater impact) are provided for CSD items (percentage who scored from 8 to 10), cough-related physical impact items (percentage who indicated frequently or mostly/always) and cough-related impact on everyday life items (percentage who indicated quite a bit, very much or an extreme amount). For the LCQ, proportions of patients who assigned the lowest scores (1 to 3, greater impact) to individual domains were calculated. To account for missing data, outcomes are calculated based on the number of patients who responded to each individual item, rather than on the total number of survey participants. Differences between RCC and UCC groups, and between men and women, were evaluated using the chi-squared test and t-test. All analyses were performed using the IBM SPSS 20.0.0 statistical program.

## Results

Of the 203 patients in 17 Spanish outpatient clinics initially identified as being eligible for participation, 196 patients were enrolled. Of the seven patients who were not enrolled, five did not provide informed consent and two were current smokers.

**FIGURE 1:**
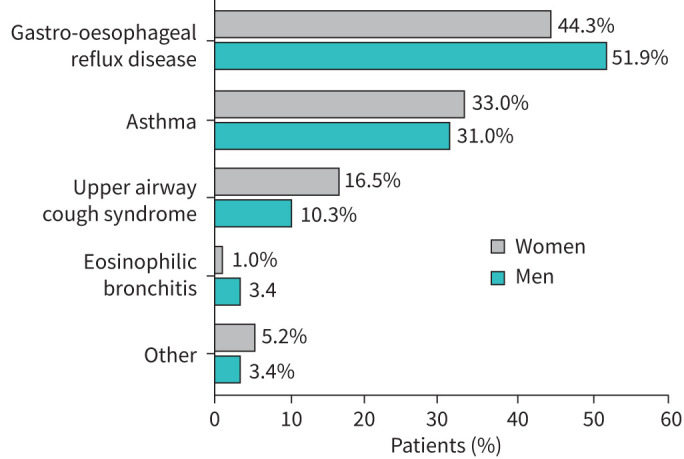
Cough-related diseases in patients with refractory chronic cough.

The enrolled population comprised 152 women (77.6%) and 44 men, of mean±sd age 58.5±13.3 years, with 166 (84.7%) enrolled from pulmonology clinics and 30 (15.3%) from allergy clinics. The diagnosis was RCC in 126 patients (64.3%) and UCC in 70 patients (35.7%); the proportion of women was similar (≈78%) in each group. Mean cough duration was 6.4±5.0 years (range 1−21). The most frequent cough-related diseases in RCC patients were GORD (46.0%) and asthma (32.5%) with no significant differences by sex (figure 1). There were no significant differences in cough characteristics (duration, frequency, type, triggers) or presence of atopy between men and women (supplementary table S1).

The printed survey was completed by 191 (97.4%) patients (149 women and 42 men; 121 with RCC and 70 with UCC), with no differences in characteristics between respondents and the overall sample. Some items, such as those related to work, childcare or relationships, may not have been applicable to all patients.

### Cough severity as perceived by patients

The mean cough severity score on the 0–100 VAS was 62.9±20.0 mm and was slightly higher in RCC than UCC patients (65.2 *versus* 59.0 mm, p*=*0.039), but similar between men and women (61.2 *versus* 63.4 mm, p*=*0.543).

The proportion of patients indicating scores of 8 to 10 for cough characteristic items assessed by the CSD did not differ significantly between RCC and UCC groups but was higher in women than men for the perception of harshness (p*=*0.016), physical discomfort (p*=*0.011) and disruption in sleep due to cough (not statistically significant: p*=*0.064) (table 1).

**TABLE 1:** Proportion of patients who indicated scores of 8 to 10 (high severity/impact) for cough characteristic items of the adapted Cough Severity Diary

**Parameter**	**Cough diagnosis**				**Sex**		
	**All**	**RCC**	**UCC**	**p-value (RCC *versus* UCC)**	**Men**	**Women**	**p-value (men *versus* women)**
**Patients n**	191	121	70		42	149	
**Cough frequency: from 0 (never) to 10 (constantly)**	45 (23.7)	32 (26.7)	13 (18.6)	0.205	8 (19.0)	37 (25.0)	0.423
**Cough episodes: from 0 (never) to 10 (always)**	39 (20.6)	25 (21.0)	14 (20.0)	0.869	6 (14.3)	33 (22.4)	0.249
**Urge to cough: from 0 (never) to 10 (constantly)**	60 (31.7)	42 (35.3)	18 (25.7)	0.172	11 (26.6)	49 (33.1)	0.445
**Harsh cough: from 0 (not at all) to 10 (extremely)**	51 (27.0)	35 (29.4)	16 (22.9)	0.327	5 (12.2)	46 (31.1)	0.016
**Physical discomfort due to cough: from 0 (none) to 10 (extreme)**	53 (28.0)	38 (31.9)	15 (21.4)	0.121	5 (12.2)	48 (32.4)	0.011
**Disruption in activities due to cough: from 0 (not at all) to 10 (could not perform activities)**	34 (18.0)	26 (21.8)	8 (11.4)	0.072	6 (14.6)	28 (18.9)	0.527
**Disruption in sleep due to cough: from 0 (not at all) to 10 (could not sleep at all)**	33 (17.6)	24 (20.3)	9 (13.0)	0.207	3 (7.5)	30 (20.4)	0.064

Data are presented as n (%) unless otherwise indicated. Results pertained to the day before the study visit. RCC: refractory chronic cough; UCC: unexplained chronic cough.

### Quality of life

The mean LCQ total score was 11.9±3.5 and was slightly lower in patients with RCC than UCC. There were no significant differences between RCC and UCC groups in individual domain scores (table 2). Compared with men, women had a significantly lower mean LCQ total score (11.6 *versus* 13.0, p*=*0.020) and lower mean scores for the physical (p*=*0.004), psychological (p*=*0.058) and social (p*=*0.071) domains of the LCQ (table 2), indicating poorer cough-related QoL. Women also had significantly lower scores than men for 11 of the 19 individual LCQ items, among them tiredness, interference with job or daily tasks, and disturbed sleep (supplementary table S2). The proportion of women *versus* men who indicated Likert scores of 1 to 3 for individual LCQ items was significantly greater for tiredness (p*=*0.036), embarrassment (p*=*0.049), interference with overall enjoyment of life (p*=*0.048), energy level (p*=*0.032) and interruption of conversation or telephone calls (p*=*0.009) (supplementary table S3.)

**TABLE 2:** Leicester Cough Questionnaire scores

**Leicester Cough Questionnaire**	**All**	**Cough diagnosis**			**Sex**		
		**RCC**	**UCC**	**p-value (RCC *versus* UCC)**	**Men**	**Women**	**p-value (men *versus* women)**
**Patients n**	191	121	70		42	149	
**Physical domain score**	4.27±1.12	4.16±1.12	4.47±1.10	0.068	4.72±1.08	4.16±1.10	0.004
**Psychological domain score**	3.81±1.32	3.76±1.34	3.91±1.28	0.435	4.15±1.28	3.72±1.31	0.058
**Social domain score**	4.11±1.44	4.01±1.47	4.28±1.37	0.221	4.47±1.33	4.01±1.46	0.071
**Total score**	11.90±3.53	11.68±3.57	12.29±3.44	0.245	13.02±3.46	11.59±3.49	0.020

Data are presented as mean±sd unless otherwise indicated. Score interpretation: physical, psychological, and social domain score from 1 to 7, and total score ranges from 3 to 21. Lower scores indicate poorer cough-related quality of life. RCC: refractory chronic cough; UCC: unexplained chronic cough.

### Impact of cough on patients’ everyday life

The proportion of patients in the overall sample who responded “frequently” or “mostly/always” for cough-related physical impact items was highest for “feeling unable to speak fluently” (58.0%), followed by feeling tired/drained (46.6%), interference with eating (need to eat slowly or stop for a while; 35.4%), stress urinary incontinence (31.7%), and feeling breathless or wheezy (28.0%) (table 3). Patients with RCC reported “feeling breathless or wheezy” more frequently than UCC patients, but there were no between-group differences in other physical items. Significantly higher proportions of women than men indicated “frequently” or “mostly/always” for the cough-related physical impact items of feeling tired/drained (51.7% *versus* 28.6%, p*=*0.008), feeling unable to speak fluently (62.3% *versus* 42.9%, p*=*0.024), interference with eating (40.1% *versus* 19.0%, p*=*0.012) and cough-related stress urinary incontinence (38.1% *versus* 9.5%, p<0.001).

**TABLE 3:** Proportion of patients who reported “frequently” or “mostly/always” to cough-related physical impact items

**Physical item**	**All**	**Cough diagnosis**			**Sex**		
		**RCC**	**UCC**	**p-value (RCC *versus* UCC)**	**Men**	**Women**	**p-value (men *versus* women)**
**Patients n**	191	121	70		42	149	
**Cough makes patient feel drained or tired (n=189)**	88 (46.6)	39 (51.3)	27 (38.6)	0.091	12 (28.6)	76 (51.7)	0.008
**Cough makes patient feel breathless or wheezy (n=189)**	53 (28.0)	41 (34.5)	12 (17.1)	0.011	11 (26.2)	42 (28.6)	0.762
**Cough makes patient faint (n=188)**	4 (2.1)	4 (3.4)	0 (0.0)	0.299	1 (2.4)	3 (2.1)	1.000
**Cough makes patient unable to speak fluently (n=188)**	109 (58.0)	73 (61.3)	36 (52.2)	0.219	18 (42.9)	91 (62.3)	0.024
**Cough interferes with meals (need to eat slowly or stop eating for a while) (n=189)**	67 (35.4)	44 (37.0)	23 (32.9)	0.568	8 (19.0)	59 (40.1)	0.012
**Cough provokes urinary incontinence (urinary loss) (n=189)**	60 (31.7)	39 (32.8)	21 (30.0)	0.692	4 (9.5)	56 (38.1)	<0.001

Data are presented as n (%) unless otherwise indicated. For these items, patients were instructed to provide an assessment not limited to a specific time period but considering how their cough had been impacting their lives in general. Not all patients responded to all items. The number of respondents is specified for each item. Percentages are calculated according to the number of respondents. Response options were: 1) Never/hardly ever; 2) Rarely; 3) Sometimes; 4) Frequently; 5) Mostly/always. RCC: refractory chronic cough; UCC: unexplained chronic cough.

Overall, 57.1% of patients indicated that cough impacted “quite a bit/much/very much” on their QoL (table 4). By item, the greatest cough-related impact on everyday life (≥30% of the sample) was on mood or emotions (41.9%), followed by physical activity (37.0%), everyday activities (36.6%), sleep (34.6%), sports/hobbies and leisure time (32.8%) and sleep of closer relatives (30.3%). The proportion of patients who reported “quite a bit/much/very much” impact on everyday life was significantly higher in the RCC *versus* UCC group for sleep (p*=*0.010), work productivity (p*=*0.041) and capacity to perform activities that require concentration (p*=*0.028). A significantly higher proportion of women than men reported “quite a bit/much/very much” cough-related impact on QoL in general (61.1% *versus* 42.9%, p*=*0.035). Proportions of women were higher than men for several other items of cough-related impact on everyday life but without reaching statistical significance (table 4).

**TABLE 4:** Proportion of patients who reported “quite a bit/very much/extremely high” to items of cough-related impact on everyday life

**Everyday life item**	**All**	**Cough diagnosis**			**Sex**		
		**RCC**	**UCC**	**p-value (RCC *versus* UCC)**	**Men**	**Women**	**p-value (men *versus* women)**
**Patients n**	191	121	70		42	149	
**Cough impacts patient's quality of life (n=191)**	109 (57.1)	72 (59.5)	37 (52.9)	0.371	18 (42.9)	91 (61.1)	0.035
**Cough impairs patient's sleep (n=191)**	66 (36.4)	50 (41.3)	16 (22.9)	0.010	10 (23.8)	56 (37.6)	0.097
**Cough affects patient's mood or emotions (n=191)**	80 (41.9)	49 (40.5)	31 (44.3)	0.609	15 (35.7)	65 (43.6)	0.359
**Cough makes patient feel anxious or depressed (n=191)**	54 (28.3)	32 (26.4)	22 (31.4)	0.461	12 (28.6)	42 (28.2)	0.961
**Cough affects everyday activities (*i.e.*, work, children or relatives care, householders) (n=191)**	70 (36.6)	48 (39.7)	22 (31.4)	0.255	13 (31.0)	57 (38.3)	0.386
**Cough has conditioned patient's professional development (difficulties in finding a suitable job, need more time to do work due to cough) (n=181)**	32 (17.7)	24 (21.6)	8 (11.4)	0.080	5 (12.2)	27 (19.3)	0.295
**At work, patient needs extra pauses or works slower due to cough (n=124)**	19 (15.3)	16 (20.5)	3 (6.5)	0.041	4 (13.8)	15 (15.8)	1.000
**Patient has been in sick leave due to cough (with no other concurrent disease) (n=164)**	19 (11.6)	10 (9.8)	9 (14.5)	0.361	3 (8.1)	16 (12.6)	0.569
**Cough affects patient's relationship with others (*i.e.*, close friends, relatives) (n=189)**	44 (23.3)	27 (22.7)	17 (24.3)	0.802	7 (17.1)	37 (25.0)	0.288
**Cough limits hobbits or leisure (going to the cinema, theatre, restaurants) (n=189)**	62 (32.8)	35 (29.4)	27 (38.6)	0.195	11 (26.8)	51 (34.5)	0.357
**Cough limits patient's capacity to make some sport or physical activity (n=189)**	70 (37.0)	48 (40.3)	22 (31.4)	0.221	14 (34.1)	56 (37.8)	0.665
**Cough limits patient's capacity to perform activities requiring concentration, like driving or riding a bike (n=187)**	40 (21.4)	31 (26.5)	9 (12.9)	0.028	5 (12.2)	35 (24.0)	0.104
**Patient's cough affects the quality of life of closer relatives (*i.e.*, spouse, family) (n=188)**	52 (27.7)	36 (30.5)	16 (22.9)	0.257	12 (29.3)	40 (27.2)	0.795
**Patient's cough affects the sleep of closer relatives (n=185)**	56 (30.3)	40 (34.5)	16 (23.2)	0.106	13 (31.0)	43 (30.1)	0.913
**Cough affects patient's caring of his/her children (n=149)**	28 (18.8)	20 (22.0)	8 (13.8)	0.212	7 (21.9)	21 (17.9)	0.614

Data are presented as n (%) unless otherwise indicated. For these items, patients were instructed to provide an assessment not limited to a specific time period but considering how their cough had been impacting their lives in general. Not all patients responded to all items. The number of respondents is specified for each item. Percentages are calculated according to the number of respondents. Response options were: 1) Not at all; 2) Slightly; 3) Somewhat; 4) Moderately; 5) Quite a bit; 6) Very much; 7) Extremely high. RCC: refractory chronic cough; UCC: unexplained chronic cough.

## Discussion

This study describes the perceptions of RCC/UCC patients about the impact of chronic cough on their QoL and aspects of everyday life. To the best of our knowledge, this is the first study to explore the impact of cough in Spanish patients with RCC or UCC from outpatient hospital clinics. The results indicate that patients with RCC and UCC experience a substantial and similar disease burden. In several aspects, women perceived a more negative impact of cough than men.

In this study, the impact of chronic cough on QoL and various aspects of patients’ everyday life was similar regardless of the underlying diagnosis (RCC or UCC), suggesting that these entities behave similarly, and that chronic cough is the main driver of impairment. Although minor differences found between groups with RCC and UCC could be due to an associated disease, this cannot be confirmed by the present study. The 2020 European Respiratory Society guidelines uses the term chronic refractory cough to refer to patients in whom a diagnostic workup has been performed, and cough is refractory to conventional treatment of cough-associated phenotypes or traits [9]. Recent interpretations of the chronic cough phenomenon suggest a common underlying pathophysiology with aberrant neurophysiology (the cough hypersensitivity syndrome) where chronic cough is a major presentation regardless of the associated condition, if any exists [12].

The cohort was representative of a usual RCC/UCC population attended at outpatient hospital clinics in that it was predominantly female (78%), most (64%) had RCC and the mean duration of chronic cough was >6 years. The mean score of 63 mm on the cough severity 0–100 VAS exceeded by >20 mm the ≥40 mm value accepted as the threshold to consider cough as moderate or severe and for inclusion in recent phase 3 clinical trials [31]. The mean cough severity VAS score was similar irrespective of cough classification (RCC or UCC) or sex. There were also no notable sex differences in general cough characteristics such as duration, frequency and triggers.

Overall, patients perceived that chronic cough had a substantial negative impact on QoL, interfering with sleep, affecting moods and emotions, and limiting their ability to conduct everyday home-related activities or participate in sports and social activities. However, women perceived greater detriment to their QoL than men and indicated that chronic cough exacted a greater physical and psychological toll on their everyday lives. The Korean National Health and Nutrition Examination Survey 2010‒2016 also reported significantly lower QoL scores in female *versus* male adults with chronic cough [22]. In our study, more women than men reported feeling drained or tired due to cough and experienced cough-related stress urinary incontinence. Feeling tired or drained was also reported by a high proportion of respondents (72%) in a UK survey of chronic cough, albeit without sex differences, whereas the prevalence of urinary incontinence was much higher in women than men (55% *versus* 5%) [15]. Significant differences between women and men (87 *versus* 77%) in activity limitations due to cough were reported in a European internet survey, although no significant sex-related differences were found with respect to the impact of cough on QoL [19]. The clear differences we observed between men and women in their perceptions of the impact of chronic cough on everyday life merit closer attention in terms of identifying patients suitable for treatment. In particular, the high prevalence of cough-related stress urinary incontinence in women cannot be overlooked, and the effect of cough suppressants on this complication deserves further investigation.

Chronic refractory cough is difficult to treat successfully, as the usual approach of sequentially evaluating and treating the presumed underlying cause of chronic cough has, by definition, failed in this patient subset [11]. Neuromodulators, such as opioids, gabapentanoids and tricyclic antidepressants, have shown benefits in treating neurogenic chronic cough, supporting cough hypersensitivity syndrome as an underlying mechanism in RCC and UCC [9, 12, 32, 33]. Nevertheless, a clear need exists for novel agents that target cough hypersensitivity directly, rather than by acting on traits that cause hypersensitivity.

A better understanding of the distinct pathophysiological process underlying RCC/UCC and the cough hypersensitivity syndrome has prompted investigation into targeted therapies that inhibit pathways associated with pathological cough. These include P2X3 antagonists, transient receptor potential channel antagonists, voltage-gated sodium channel blockers, neuromodulators and neurokinin-1–receptor antagonists [11]. Of the candidate agents, the P2X3 antagonist gefapixant has shown evidence of benefit in patients with RCC or UCC in phase 3 studies, including reduction of cough counts and improvement in QoL [31, 34].

The study has several limitations. As the setting was outpatient hospital clinics, the population may have included patients with more severe symptomatology and thus not reflect the wider RCC/UCC population. Conversely, this approach may offer the advantage of identifying patients who are more likely to benefit from upcoming treatments for RCC/UCC. In this study, information was collected directly from patients *via* a printed survey, without physician overview, and was not subjected to source data verification. Although a limitation in terms of data integrity, this approach has the benefit of reflecting patients’ unbiased perception of their own condition. Whereas some data were collected using the validated LCQ, information was also derived from unvalidated sources as several questions were adapted from previous studies or created *ad hoc* for this study. Furthermore, because the CSD was adapted to accommodate the timing of patients’ clinical appointments, scores were not calculated and only a description of the responses is provided. The study describes patients with RCC/UCC from outpatient hospital clinics but is limited by the lack of a control group and lack of follow-up. A longitudinal design could have provided a more complete picture of the impact of cough, as several instruments assessed only the moment that patients responded (VAS severity score), the day before (CSD) or the past 2 weeks (LCQ), not the overall impact of cough in the long term. Results of comparisons must be interpreted with caution given that the sample size was calculated to have power only to describe the sample as a whole and not by subgroups. Finally, the generalisability of some specific results in this cohort of Spanish outpatients with RCC/UCC may be limited. Nevertheless, the overall findings, which clearly indicate that RCC and UCC adversely impact patients’ QoL and everyday life, are applicable to other RCC/UCC populations.

### Conclusions

Patients with RCC and UCC experience a significant disease burden that impairs their QoL and impacts on their physical and psychological health and everyday activities. The impact is similar in RCC and in UCC, suggesting that, whether or not an associated condition or treatable trait exists, chronic cough is the main driver of impairment. As women form the larger part of the chronic cough population, clinicians should be vigilant and proactive in assessing women with chronic cough since the burden appears to be greater than that of men. Newer therapies targeting underlying disease mechanisms may address an unmet need in patients with RCC and UCC and alleviate the substantial associated disease burden.

## Supplementary material

10.1183/23120541.00425-2023.Supp1 **Please note:** supplementary material is not edited by the Editorial Office, and is uploaded as it has been supplied by the author.
Supplementary material 00425-2023.SUPPLEMENT
