# Supplementary material for: Burden of refractory and unexplained chronic cough on patients’ lives: a cohort study
Source: ERJ Open Res. 2023 Sep 25;9(5):00425-2023. doi: 10.1183/23120541.00425-2023 (PMC10518856; doi:10.1183/23120541.00425-2023)
Supplement: Supplementary file 1 [file 00425-2023.SUPPLEMENT.pdf]

Burden of Refractory and Unexplained Chronic Cough on Patients' Life: a cohort study

SUPPLEMENTARY TABLES

Supplementary Table 1 Characteristics of cough in men and women

**Characteristic**		**All (N=196)**	**Men (N=44)**	**Women (N=152)**	**p-value (men *vs* women)**
**Duration, y**	Mean (SD)	6.4 (5.0)	6.7 (5.7)	6.3 (4.8)	0.600
**Cough classification**	Refractory, n (%)	126 (64.3)	29 (65.9)	97 (63.8)	0.799
	Unexplained, n (%)	70 (35.7)	15 (34.1)	55 (36.2)	
**Cough frequency[a]**	Continuous, n (%)	156 (79.6)	33 (75.0)	123 (80.9)	0.619
	Intermittent, but not seasonal, n (%)	34 (17.3)	9 (20.5)	25 (16.4)	
	Intermittent, seasonal, n (%)	6 (3.1)	2 (4.5)	4 (2.6)	
**Cough characteristic**	Predominantly dry, n (%)	171 (87.2)	40 (90.9)	131 (86.2)	0.608
	Predominantly productive, n (%)	25 (12.8)	4 (9.1)	21 (13.8)	
**Cough triggers**	Any trigger, n (%)	62 (31.6)	18 (40.9)	44 (28.9)	0.133
	Speaking or laughing, n (%)	25 (12.8)	7 (15.9)	18 (11.8)	0.476
	Cold air or temperature change, n (%)	21 (10.7)	7 (15.9)	14 (9.2)	0.206
	Dust, pollen, or other air irritants/particles like perfumes, n (%)	22 (11.2)	6 (13.6)	16 (10.5)	0.565
	Environmental tobacco smoke, n (%)	13 (6.6)	3 (6.8)	10 (6.6)	1.000
	Agents present at work (occupational), n (%)	2 (1.0)	1 (2.3)	1 (0.7)	0.399
	Eating or a particular type of food, n (%)	17 (8.7)	4 (9.1)	13 (8.6)	1.000
	Exercise or exertion, n (%)	13 (6.6)	2 (4.5)	11 (7.2)	0.736
	Other, n (%)	14 (7.1)	3 (6.8)	11 (7.2)	1.000
**Atopy[b]**	Yes, n (%)	66 (37.7)	19 (47.5)	47 (34.8)	0.146

SD standard deviation

[a] Continuous: Patient suffers from cough every day or nearly every day. Intermittent, but not seasonal: Patient suffers periods of cough and periods of remission, but the cough is not present at specific periods in the year. Intermittent, seasonal: Patient has a chronic cough at specific times or seasons.

[b] Atopy, according to local standard tests (positive skin prick test or positive determination of serum-specific IgE to aeroallergens or foods).

Supplementary Table 2 Mean scores of the 19 individual items of the Leicester Cough Questionnaire in men and women

Leicester Cough Questionnaire items	All (N=191)[a]	Men (N=42)	Women (N=149)	p-value (men *vs* women)
	Mean (SD)	Mean (SD)		
1. In the last 2 weeks, have you had chest or stomach pains as a result of your cough?[b]	4.8 (1.5)	5.3 (1.4)	4.7 (1.5)	0.018
2. In the last 2 weeks, have you been bothered by sputum (phlegm) production when you cough?[c]	4.5 (1.9)	4.6 (1.9)	4.4 (1.9)	0.552
3. In the last 2 weeks, have you been tired because of your cough?[b]	4.1 (1.6)	4.8 (1.7)	3.9 (1.6)	0.004
4. In the last 2 weeks, have you felt in control of your cough?[d]	2.4 (1.4)	2.4 (1.3)	2.4 (1.4)	0.812
5. How often during the last 2 weeks have you felt embarrassed by your coughing?[b]	4.0 (1.9)	4.6 (1.8)	3.8 (1.9)	0.013
6. In the last 2 weeks, my cough has made me feel anxious[b]	4.6 (1.6)	5.1 (1.7)	4.4 (1.6)	0.015
7. In the last 2 weeks, my cough has interfered with my job, or other daily tasks[b]	4.6 (1.6)	5.2 (1.6)	4.5 (1.6)	0.004
8. In the last 2 weeks, I felt that my cough interfered with the overall enjoyment of my life[b]	4.4 (1.7)	4.7 (1.6)	4.2 (1.7)	0.102
9. In the last 2 weeks, exposure to paints or fumes has made me cough[b]	4.1 (2.0)	4.8 (1.9)	3.9 (2.0)	0.010
10. In the last 2 weeks, has your cough disturbed your sleep?[b]	4.5 (1.8)	5.2 (1.7)	4.3 (1.8)	0.006
11. In the last 2 weeks, how many times a day have you had coughing bouts?[e]	3.8 (1.4)	3.8 (1.5)	3.8 (1.4)	0.981
12. In the last 2 weeks, my cough has made me feel frustrated[b]	4.4 (1.8)	4.7 (1.7)	4.2 (1.8)	0.121
13. In the last 2 weeks, my cough has made me feel fed up[b]	3.4 (1.7)	3.9 (1.6)	3.2 (1.7)	0.037
14. In the last 2 weeks, have you suffered from a hoarse voice as a result of your cough?[b]	5.0 (1.7)	5.4 (1.8)	4.8 (1.7)	0.044
15. In the last 2 weeks, have you had a lot of energy?[d]	3.5 (1.6)	4.0 (1.8)	3.3 (1.5)	0.013
16. In the last 2 weeks, have you worried that your cough may indicate serious illness?[b]	4.6 (1.9)	4.7 (1.7)	4.6 (1.9)	0.602
17. In the last 2 weeks, have you been concerned that other people think something is wrong with you, because of your cough?[b]	3.6 (2.0)	3.9 (2.0)	3.5 (2.0)	0.232
18. In the last 2 weeks, my cough has interrupted conversation or telephone calls[f]	3.7 (1.7)	4.3 (1.6)	3.6 (1.7)	0.020
19. In the last 2 weeks, I feel that my cough has annoyed my partner, family or friends[g]	3.8 (1.9)	3.8 (1.8)	3.8 (2.0)	0.979

SD standard deviation

[a] Five patients (two men and three women) did not complete the Leicester Cough Questionnaire.

[b] Responses: 1=All of the time; 2=Most of the time; 3=A good bit of the time; 4=Some of the time; 5=A little of the time; 6=Hardly any of the time; 7=None of the time.

[c] Responses: 1=Every time; 2=Most times; 3=Several times; 4=Sometimes; 5=Occasionally; 6=Rarely; 7=Never.

[d] Responses: 1=None of the time; 2=Hardly any of the time; 3=A little of the time; 4=Some of the time; 5=A good bit of the time; 6=Most of the time; 7=All of the time.
[e] Responses: 1=All of the time (continuously); 2=Most times during the day; 3=Several times during the day; 4=Sometimes during the day; 5=Occasionally through the day; 6=Rarely; 7=None.
[f] Responses: 1=Every time; 2=Most times; 3=A good bit of the time; 4=Some of the time; 5=A little of the time; 6=Hardly any of the time; 7=None of the time.
[g] Responses for item 19: 1=Every time I cough; 2=Most times when I cough; 3=Several times when I cough; 4=Sometimes when I cough; 5=Occasionally when I cough; 6=Rarely; 7=Never.

Supplementary Table 3 Proportion of patients who indicated scores of 1–3 (indicating greater impairment in quality of life) to the 19 individual items of the Leicester Cough Questionnaire

**Leicester Cough Questionnaire item**	**All (N=191)[a]**	**Men (N=42)**	**Women (N=149)**	**p-value (men *vs* women)**
	**n (%)**	**n (%)**		
1. In the last 2 weeks, have you had chest or stomach pains as a result of your cough?	32 (16.8)	4 (9.5)	28 (18.8)	0.241
2. In the last 2 weeks, have you been bothered by sputum (phlegm) production when you cough?	68 (35.6)	14 (33.3)	54 (36.2)	0.728
3. In the last 2 weeks, have you been tired because of your cough?	72 (37.7)	10 (23.8)	62 (41.6)	0.036
4. In the last 2 weeks, have you felt in control of your cough?	162 (85.3)	36 (85.7)	126 (85.1)	0.926
5. How often during the last 2 weeks have you felt embarrassed by your coughing?	75 (39.3)	11 (26.2)	64 (43.0)	0.049
6. In the last 2 weeks, my cough has made me feel anxious	43 (22.9)	8 (19.5)	35 (23.8)	0.562
7. In the last 2 weeks, my cough has interfered with my job, or other daily tasks	42 (22.3)	7 (17.1)	35 (23.8)	0.360
8. In the last 2 weeks, I felt that my cough interfered with the overall enjoyment of my life	60 (32.3)	8 (19.5)	52 (35.9)	0.048
9. In the last 2 weeks, exposure to paints or fumes has made me cough	70 (37.6)	10 (25.0)	60 (41.1)	0.063
10. In the last 2 weeks, has your cough disturbed your sleep?	55 (29.3)	8 (19.5)	47 (32.0)	0.121
11. In the last 2 weeks, how many times a day have you had coughing bouts?	100 (53.2)	20 (48.8)	80 (54.4)	0.522
12. In the last 2 weeks, my cough has made me feel frustrated	67 (35.6)	11 (26.8)	56 (38.1)	0.183
13. In the last 2 weeks, my cough has made me feel fed up	97 (51.9)	16 (39.0)	81 (55.5)	0.062
14. In the last 2 weeks, have you suffered from a hoarse voice as a result of your cough?	43 (23.2)	7 (17.1)	36 (25.0)	0.289
15. In the last 2 weeks, have you had a lot of energy?	107 (58.5)	18 (43.9)	89 (62.7)	0.032
16. In the last 2 weeks, have you worried that your cough may indicate serious illness?	48 (26.1)	9 (22.0)	39 (27.3)	0.494
17. In the last 2 weeks, have you been concerned that other people think something is wrong with you, because of your cough?	94 (50.5)	18 (43.9)	76 (52.4)	0.336
18. In the last 2 weeks, my cough has interrupted conversation or telephone calls	83 (44.6)	11 (26.8)	72 (49.7)	0.009
19. In the last 2 weeks, I feel that my cough has annoyed my partner, family or friends	94 (50.5)	17 (41.5)	77 (53.1)	0.188

[a] Five patients (two men and three women) did not complete the Leicester Cough Questionnaire. See Supplementary Table 2 for the description of scores.
